# Revisiting the Age-Prospective Memory Paradox Using Laboratory and Ecological Tasks

**DOI:** 10.3389/fpsyg.2021.691752

**Published:** 2021-06-17

**Authors:** Yu Wen Koo, David L. Neumann, Tamara Ownsworth, David H. K. Shum

**Affiliations:** ^1^School of Applied Psychology, Griffith University, Mt Gravatt, QLD, Australia; ^2^Menzies Health Institute of Queensland, Griffith University, Gold Coast, QLD, Australia; ^3^Department of Rehabilitation Sciences, The Hong Kong Polytechnic University, Kowloon, Hong Kong

**Keywords:** prospective memory, aging, age-PM paradox, older adults, young adults

## Abstract

Prospective memory (PM) is the ability to perform a planned action at a future time. Older adults have shown moderate declines in PM, which are thought to be driven by age-related changes in the prefrontal cortex. However, an age-PM paradox is often reported, whereby deficits are evident in laboratory-based PM tasks, but not naturalistic PM tasks. The key aims of this study were to: (1) examine the age-PM paradox using the same sample across laboratory and ecological settings; and (2) determine whether self-reported PM and cognitive factors such as working memory and IQ are associated PM performance. Two PM tasks were administered (ecological vs. laboratory) to a sample of 23 community-dwelling older adults (*M_age_* = 72.30, *SD_age_* = 5.62) and 28 young adults (*M_age_* = 20.18, *SD_age_* = 3.30). Participants also completed measures of general cognitive function, working memory, IQ, and self-reported memory. Our results did not support the existence of the age-PM paradox. Strong age effects across both laboratory and ecological PM tasks were observed in which older adults consistently performed worse on the PM tasks than young adults. In addition, PM performance was significantly associated with self-reported PM measures in young adults. For older adults, IQ was associated with time-based PM. These findings suggest that the age-PM paradox is more complex than first thought and there are differential predictors of PM performance for younger and older adults.

## Introduction

Despite our best intentions, we sometimes, and often fail to remember to perform an intended action on the appropriate occasion or at the right time. Remembering to carry out an intended action in the future is called prospective memory (PM). Failing to remember to carry out future intentions has functional and clinical relevance because it is related to independence, quality of life, and everyday functioning (Woods et al., 2012, 2014, 2015). PM tasks can be classified as event-based, which the execution of the intended action is initiated in response to a particular target event or cue (e.g., posting a letter when passing a post box), or time-based, which require an individual to remember to perform the intended action at a specific time or after a specified period of time has elapsed (e.g., remembering to check a message in 15 min or remembering to turn off the stove in 30 min). The present study examined age-related differences in PM as measured by a number of tasks and the relationship between individual difference variables and PM performance in a sample of young and older adults.

Prospective memory performance changes with aging. Aging typically comes with a deterioration of cognitive functioning, such as memory (i.e., working memory and episodic memory; Luo and Craik, 2008), and executive functions (Fisk and Sharp, 2004). This decline is associated with substantial shrinkage of gray and white matter in the prefrontal cortex, hippocampus, basal ganglia and changes in structural connectivity (Raz et al., 2005). Consequently, cognitive processes such as learning, memory (including PM), and executive functions that rely on the prefrontal and medial temporal cortex functions decline with age (Burke and Barnes, 2006). Previous research has also shown that older adults’ ability to perform tasks independently in the home and in the community is dependent on cognitive processes such as executive functions (Dodge et al., 2006; Royall et al., 2007). Thus, understanding PM processes in older adulthood has implications for individuals’ everyday functioning and quality of life.

Prospective memory and aging research has shown conflicting age effects, dubbed the age-PM paradox. That is, an absence of age-related decrease in PM performance if PM is assessed in a naturalistic setting (i.e., daily life) but the presence of PM deficits when assessed with laboratory-based tasks (Henry et al., 2004; Uttl, 2008; Kliegel et al., 2016). This pattern is unique because in many other cognitive domains, increasing task familiarity (Kliegel et al., 2007), or measuring performance in the context of daily life (Phillips et al., 2008), only attenuate, rather than eliminate, age-related effects on performance.

It has been postulated that older adults show the greatest age-related decline on laboratory-based PM tasks because these tasks require greater prefrontal cortex involvement. However, older people’s naturalistic PM task performance is preserved because they are routine behaviors and rely on more automatic cognitive processes, minimizing the use of attentional and executive resources (McDaniel and Einstein, 2007). Consequently, older adults are more vulnerable to PM failures on tasks with high strategic and novel demands (i.e., self-initiated executive control of monitoring and cue detection; Craik et al., 1986). Some other explanations for these differential age-effects include variations in the complexity or cognitive demands of the ongoing task (Schnitzspahn et al., 2011), familiarity and experience (Altgassen et al., 2010), differences in daily demands, motivation (Rendell and Craik, 2000); social importance (Niedzwienska et al., 2013); incentives for young adults (Aberle et al., 2010); and use of external aids (i.e., such as diaries/alarms) in naturalistic settings (Moscovitch, 1982; Ihle et al., 2012; Altgassen et al., 2015; Haines et al., 2020).

However, a critical evaluation of the evidence for the age-PM paradox suggests that more research is required to confirm that older individuals are not impaired on naturalistic PM tasks. Two main limitations were identified after reviewing the literature. First, this paradox is mostly inferred from results of studies that have examined the effect of age on laboratory and naturalistic tasks separately. That is, relatively few studies have concurrently examined PM in laboratory and naturalistic settings within the same study and comparing the same samples (Rendell and Craik, 2000; Bailey et al., 2010; Schnitzspahn et al., 2011, 2018; Niedzwienska and Barzykowski, 2012; Kvavilashvili et al., 2013; Haines et al., 2020). Therefore, these age effects need careful investigation using the same sample in both naturalistic and laboratory settings.

Second, the typical naturalistic tasks are usually one-off, unreliable tasks that require little effort to perform (e.g., calling experimenter at a specified time, asking for a belonging back, sending a postcard; Kvavilashvili et al., 2013). As an exception, Bailey et al. (2010) embedded a “classic,” event PM task within a naturalistic ongoing task. Participants completed a questionnaire on their digital organizers in response to random alarms (naturalistic PM task), while the “classic” laboratory task was to respond to items that were presented with an upper case. They found that the performance advantage for older adults was only observed within the naturalistic task, where older adults were prompted to complete a questionnaire during their everyday lives. Bailey et al. (2010) concluded that older adults’ ability to compensate and further outperform their younger counterparts in typical naturalistic studies may be due, at least in part, to the nature of the ongoing tasks typical to their everyday lives.

Although not directly investigating the age-PM paradox, Shum et al. (2013) showed that event- and time-based PM tasks can be measured using a complex ecological PM paradigm without compromising experimental control. Their PM paradigm required participants to sit at the kitchen table (in a simulated home environment), while carrying out embedded time- and event-based PM tasks (pausing a video player every 5 min and placing a white sticky dot on the top right-hand corner of any recipes that contained dairy products). As an ongoing task, young and older adults were required to use a recipe book and a price catalog to decide which recipes were the most time and cost-effective as the ongoing task. They found that younger adults outperformed older adults on both time- and event-based PM tasks. However, Shum et al. (2013) did not administer an experimental PM task to their participants in this study. Therefore, their results could not shed light on the age-PM paradox.

Most recently, Haines et al. (2020) examined time-based PM tasks across naturalistic and laboratory-based PM tasks (Virtual Week; Rendell and Craik, 2000) across three experiments. In Experiment 1, participants completed tasks individually in a laboratory, followed by 6 days of naturalistic testing. On the laboratory time-interval tasks, older adults underperformed relative to young adults, while on naturalistic time-of-day tasks, older adults outperformed young adults. Overall, they replicated the age-PM paradox (Experiment 1), but this effect was attenuated when external aids were permitted in the naturalistic task (Experiments 2 and 3). The authors concluded that a key explanation for the age-PM paradox is a lack of parallel PM task types across settings. That is, the task types systematically differ in the level of environmental cues. Importantly, these task characteristics interact with age-related changes in cognitive processes because there is more reliance on automatic rather than effortful processes by older adults. However, their naturalistic task permitted use of external aids, while also spanning across the participants’ daily lives outside of the laboratory. This does not account for differences in the ongoing daily life tasks of the participants or enable fair comparisons for those who did not use external aids. The authors also noted that older adults were using external aids to their advantage (Haines et al., 2020).

This study aimed to address the above key limitations associated with the study of the age-PM paradox. First, we administered PM tasks (both ecological and laboratory PM tasks) to the same sample, since few existing studies have investigated the age-PM paradox in this manner (Schnitzspahn et al., 2011; Kvavilashvili et al., 2013; Niedzwienska et al., 2013). Second, unlike previous naturalistic PM tasks that use single or small number of measures, our ecological PM task (adapted from Shum et al., 2013) was more complex and reliable but familiar to both age groups, while also being conducted in a controlled environment without use of external aids.

Lastly, we included self-reported PM measures to assess an individual’s subjective perceptions of their PM abilities, in addition to behavioral PM performance. Importantly, studies using self-reported PM measures have found no age-related PM declines when comparing old and young adults (e.g., Smith et al., 2000; Crawford et al., 2003), and no association between self-reported and objective PM performance (Zeintl et al., 2006). Others have also found that among older adults who reported problems with instrumental activities in daily life, higher self-reported PM failures were significantly associated with lower quality of life (Woods et al., 2015). Therefore, it is important to assess the relationship between the age-PM paradox and self-reported PM. We also investigated the ecological and convergent validity of the behavioral PM tasks and self-reported PM measures. In other words, whether self-reported PM processes in daily life are predictive of the PM task performance.

Assuming that there is an age-PM paradox, it was hypothesized that: (1) older adults would perform worse than younger adults in the laboratory PM task; but older adults would perform better than young adults in the complex ecological task; (2) both younger and older adults would perform better on event-based than time-based PM tasks; (3) there would be a significant relationship between different self-reported PM measures (i.e., convergent validity); (4) the self-reported PM measures would reflect the behavioral differences between the age groups (i.e., ecological validity), whereby older adults’ self-reported PM would be significantly associated with their objective PM performance; and (5) that cognitive measures [IQ and letter-number sequencing (LNS)] and self-reported PM measures would be associated with PM performance.

## Materials and Methods

### Participants

A sample of 58 adults [30 young adults (*M_age_* = 20.00, *SD_age_* = 3.34, 67% females), 28 older adults (*M_age_* = 71.39, *SD_age_* = 5.55, 71% females)] participated in this study. In terms of the general inclusion criteria, all participants were native English speakers, had normal or corrected-to-normal vision, and had no history of neurological or psychiatric disorders. Young adults (age range: 17–31 years) were undergraduate university students who received course credit for their participation. Healthy older adults (65–85 years) were recruited from retirement villages in the general community. The initial screening process was conducted using the Telephone Interview for Cognitive Status-Modified (TICS-M; Brandt et al., 1988). Inclusion criteria for older adults were TICS-M score > 31 (Knopman, 2010), well-preserved general cognitive functioning [mini-mental state examination (MMSE) > 25; Woods et al., 2014]. The exclusion criteria were that there was no history of neurological illness or brain injury, current or history of psychiatric illness, no current or history of alcohol or substance abuse, and no significant uncorrected visual or hearing impairment.

### Measures

#### The Telephone Interview for Cognitive Status Modified

This was used to screen older adults over the telephone prior to home visitations. The TICS-M is a brief 13-item test of cognitive functioning with scores ranging from 0 to 50. TICS-M is as reliable and valid as face-to-face administration and has a sensitivity of 94% and specificity of 100% for distinguishing normal controls from individuals with dementia (Brandt et al., 1988), and normal controls from those with mild cognitive impairment and dementia (Knopman, 2010). Test-retest reliability of TICS-m has been demonstrated to be excellent from 1 to 6 weeks (*r* = 0.96) for patients with Alzheimer’s disease (Brandt et al., 1988; Welsh et al., 1993).

#### Mini-Mental State Examination

This is a measure of general cognitive functioning commonly for older adults (Folstein et al., 1975). The difference between this measure and TICS-M is that it included visual stimuli as well as a motor task, which was important for the PM tasks. This scale includes 11 questions and requires 5–10 min to administer. It focuses on the cognitive aspects of mental functions and is divided into two sections. The first covers orientation, memory and attention, and requires vocal responses only. The second section assesses ability to name, and follow verbal and written commands. Scores range from 0 to 30. A score of 24 and higher indicates that individuals are cognitively intact, meanwhile scores of 23 and lower are indicative of cognitive impairment. Test-retest reliability is excellent for the 1-week test-retest scores (*r* = 0.90–0.97), and high internal consistency (Cronbach’s *a* > 0.80) has been demonstrated (Pangman et al., 2000).

#### Wechsler Abbreviated Scale of Intelligence

The Wechsler Abbreviated Scale of Intelligence (WASI-II) – second edition (Wechsler, 2011) is a short form IQ test designed to estimate intelligence and cognitive ability in adults and older adolescents (ages 16–89 years). In this study, the two subscales Vocabulary and Matrix Reasoning measuring crystallized and fluid intelligence were used to estimate full scale intellectual quotient from wechsler abbreviated scale of intelligence-II (FSIQ-2). In an adult sample, the average internal consistency reliability coefficients for FSIQ-2 are 0.96. The average internal consistency reliabilities for the two subtests are 0.94 for Vocabulary and Matrix Reasoning, with test-retest reliability at 0.88 for the FSIQ-2.

#### Letter-Number Sequencing Subtest

The Letter-Number Sequencing (LNS) is a subtest of the Wechsler Memory Scale–III (Wechsler, 1997) that measures working memory. Participants were presented with a series of numbers and letters, and are asked to recall the numbers in numerical order followed by the letters in alphabetical order. A series of alternating numbers and letters at the rate of about one per second was orally presented. The test begins with series of two items (one number and one letter) and continues to a maximum of eight items (four numbers and four letters). Participants were given three trials at each series length and continued until all three trials of a series length are failed. The maximum possible score for LNS is 21. Test-retest reliability was found to be between 0.71 and 0.77 (Wechsler, 2008), with high internal consistency of 0.85 (Gold et al., 1997).

### Self-Reported PM Measures

#### Prospective and Retrospective Memory Questionnaire

The Prospective and Retrospective Memory Questionnaire (PPMQ; Smith et al., 2000) is a 16-item questionnaire developed to measure the frequency of prospective (PM) and retrospective (RM) memory failures in everyday life. Eight questions measure PM (e.g., Do you decide to do something in a few minutes’ time and then forget to do it?), and eight questions measure RM failures (e.g., Do you forget what you watched on TV the previous day?). Participants are required to rate how often each type of memory failure happens in their everyday life on a five-point scale ranging from *never* (1) to *very often* (5). The reliability of the PMRQ as measured by internal consistency was acceptable (*a* = 0.86). A score for PM and RM in addition to total score can be calculated by totaling questions for each subscale with higher scores indicating more frequent everyday PM and RM failures.

#### The Brief Assessment of Prospective Memory

The Brief Assessment of Prospective Memory (BAPM; Man et al., 2011) is a 16-item self-report questionnaire designed to assess the frequency of PM failures for individuals with traumatic brain injury. Participants were required to rate their PM forgetting in the last month on a five-point scale from 1 (*never*), 2 (*rarely*), 3 (*occasionally*), 4 (*often*), 5 (*very often*), or NA (*not applicable*). The ratings were made for each of eight items to do with instrumental activities of daily living (IADL) such as managing finances, shopping, meal preparation, and eight items involving basic activities of daily living (BADL) such as eating, dressing, and personal grooming. Part A is a 16-item self-reported questionnaire that assesses PM failures within the last month. Three scores are calculated from this scale – the total overall PM, BADL subscale and IADL subscale scores. The average score is found for the total and each subscale. Part B contains the same questions but asks the participant to rate “how much of problem would it be if you did forget to complete the task.” Similar to Part A, three scores are calculated from this scale: the overall total PM score, BADL and IADL scores. For all subscales, a lower score indicates better functioning.

### PM Tasks

#### Lexical Decision Task

This was developed based on dual-task paradigm of Einstein and McDaniel (1990). For the ongoing task, participants were presented with a series of words and non-words, and were asked to judge whether they are words or non-words (pressing “F” for words, and “J” for non-words). There were 206 stimuli (97 words and 97 non-words) and 12 PM cues. For each trial, a fixation cross was presented in the center of the screen for 1,000 ms, followed by a stimulus which could either be a word, a non-word, or a PM cue displayed for 1,000 ms. A colored border appeared in every trial around the word, there were a total of 10 colors (cyan, lime green, blue, pink, gray, yellow, orange, gray, and red). For the PM task participants were asked to press the “K” key when they saw a red border background around the word. The 12 PM targets were presented in a pseudo-randomized order during the task (i.e., trials 15, 22, 29, 36, 50, 107, 121, 132, 161, 168, 183, and 187). The outcome PM measure was the proportion of correct responses. All participants were asked to describe the requirements of the PM task to ensure that they understood them prior starting.

#### Ecological PM Task

The ecological PM task was adapted from Shum et al. (2013) and included event-based and time-based components. For the ongoing task, participants were instructed to sit at a mock kitchen table to use a recipe book containing 10 recipes and a grocery catalog. Participants were asked to calculate the total cost of each recipe, working from the first page to the last page. For the event-based PM task, the participants were required to place a sticky note on recipes that are free from dairy, eggs, and meat (including fish). The explanation for bookmarking dairy-free recipes was that one of the guests coming to dinner may be allergic to dairy products. Four of these targets were placed at fixed intervals through the book. A proportion correct score was calculated based on the number of correct recipes bookmarked. For example, if three were correctly marked, a score of 75% was awarded. If the participant turned the page and moved on without bookmarking a target recipe, it was marked as missed due to no action being carried out. The maximum score for this task is 100%. The time-based PM task required participants to check the computer tablet (swipe up, to unlock) at certain time intervals using a kitchen timer placed slightly to the side just out of direct vision of the participant. The timer counted from 0:00 until the end of the task. Participants were to unlock the tablet at 8 min, and then every 7 min after that (15, 22, 29, etc.) until the task was completed. An average (rather than a total) score based upon each participant’s overall performance was used as the dependent variable because the number of opportunities varied among participants depending on how long they took to complete the ongoing task. Participants were given these instructions at the beginning of this task. Those who carried out the PM action within 15 s before or after the expected time were scored as a “hit” and those who carried out the action outside of that 15 s window were scored as “missed.” The number of time-based tasks correctly performed were scored as proportion correct as this ecological task length varies for each individual. The maximum score for this task is 100%.

### Procedure

To aid in the recruitment of participants, the older adults were assessed in their own homes if requested, while young adults participated in a room on the university campus. All participants gave their written consent prior to taking part in the study, and the study was approved by the institution ethics committee. Older adults were screened *via* telephone prior to the in-person visits (96% opted for home visits). Participants completed tasks in a counterbalanced order, of either the recipe task or lexical decision task (LDT), followed by FSIQ-2, LNS, BAPM, and PRMQ. After that, they completed the PM tasks. The study took approximately 1.5 h. All participants were able to recall the PM task instructions.

### Statistical Analyses

All data were analyzed using SPSS 25. All continuous variables followed a normal distribution with kurtosis and skewness values between −1.5 and +1.5. The data were screened for accuracy, missing values, outliers, and normality. Inferential analyses were conducted using independent samples *t*-tests to investigate differences between young and old age groups on demographic and cognitive variables. For all statistical analyses *α* was set at 0.05. Corrected degrees of freedom were used in comparisons with unequal variance. Pearson’s correlation was used to investigate relationships between cognitive variables, self-reported PM measures and objective PM performance. ANOVAs were conducted to investigate age-effects on the PM tasks.

## Results

Two young adults and five older adults from the original sample was excluded from analyses due to failure to score at least 50% proportion correct on the ongoing task on both LDT and ecological PM task. Thus, data from 23 neurologically healthy older adults (69% females, *M_age_* = 72.30, *SD_age_* = 5.62) and 28 healthy young adults (70% females, *M_age_* = 20.18, *SD_age_* = 3.39) were analyzed for the current study. Nevertheless, no older adults were excluded based on cognitive screening scores indicating that all participants were cognitively healthy. As seen in Table 1 older adults had significantly higher IQ than the young adults. For older adults, one univariate outlier was found for full scale intellectual quotient (FSIQ), one for PRMQ RM, two for LDT PM accuracy, four for time-based PM, three for event-based PM and these were removed from the analyses. For young adults, one univariate outlier was found for BAPM A total, one for LDT PM accuracy, three for time-based PM accuracy and two for event-based PM accuracy and these were removed from the analyses (Table 2).

**Table 1:** Mean scores and SDs of cognitive measures for young adults (*n* = 28) and older adults (*n* = 23).

	Young adults	Older adults	*t*	*df*	*p*	*d*
	*M (SD)*	*M (SD)*				
Age	20.18 (3.39)	72.30 (5.62)				
TICS-M		34.61 (3.12)				
MMSE		29.74 (0.45)				
Education (years)	12.71 (1.56)	14.48 (3.98)	−1.84	27.59	0.10	0.58
LNS	10.25 (2.55)	11.87 (2.22)	1.97	49	0.06	0.68
FSIQ-2	103.00 (9.83)	109.55 (9.82)	−2.34	48	0.02^*^	0.67

TICS-M, the telephone interview for cognitive status modified; MMSE, mini-mental state examination; FSIQ-2, full scale intellectual quotient from wechsler abbreviated scale of intelligence-II; and LNS, letter number sequencing.

* *p* < 0.05.

**Table 2:** Descriptive and inferential statistics for self-reported data in young adults (*n* = 28) and older adults (*n* = 23).

	Young adults	Older adults	*t*	*df*	*p*	*d*
	*M (SD)*	*M (SD)*				
**BAPM part A**						
BADL	1.59 (0.44)	1.19 (0.20)	4.24	39.26	0.00^***^	1.17
IADL	2.25 (0.62)	1.75 (0.57)	3.00	49	0.01^**^	0.84
Total	1.86 (0.41)	1.47 (0.35)	3.55	48	0.00^***^	1.02
**BAPM part B**						
BADL	2.47 (0.67)	2.98 (0.80)	−2.49	49	<0.05^*^	0.69
IADL	2.29 (0.60)	2.76 (0.63)	−2.73	49	<0.01^**^	0.76
Total	2.36 (0.60)	2.87 (0.67)	−2.87	49	<0.01^**^	0.80
**PRMQ**						
PM	23.11 (5.14)	20.13 (3.20)	2.50	44.20	<0.05^*^	0.70
RM	19.22 (3.69)	18.91 (3.70)	0.30	47	0.77	0.08

BAPM, brief assessment of prospective memory; BADL, basic activities of daily living; IADL, instrumental activities of daily living; PRMQ, prospective and retrospective memory questionnaire; PM, prospective memory; and RM, retrospective memory.

* *p* < 0.05;

** *p* < 0.01;

*** *p* < 0.001.

### Self-Reported PM Measures

As shown in Table 2, compared to older adults, young adults reported significantly more failures in PM overall and on both subscales of BAPM Part A, with large effect sizes (*d* = 1.02). For both age groups, ratings were between the *never* to *rarely* forgetting range for all subscales. However, on Part B of the BAPM, young adults reported PM failures as significantly less problematic/important when compared to older adults (medium/large effect sizes; *d* = 0.80). The means of both groups fell between *a slight* to *moderate problem* rating range for all subscales. For the PRMQ, young adults reported significantly more PM lapses than older adults, while there were no significant differences on the RM subscale. For both age groups, means were falling between the *rarely* to *sometimes*, and *never* to *rarely* forgetting range, respectively.

Correlational analyses were conducted separately for the two groups to examine the relationships between the BAPM and the PRMQ. As shown in Table 3, the PRMQ PM and RM subscale scores were significantly correlated with BAPM Part A scores for both age groups. However, there were no significant correlations between BAPM Part B and either PMRQ subscale for either age groups. This is understandable because BAPM Part B asked about importance rather than frequencies of PM impairment.

**Table 3:** Correlations between PM predictors and PM performance.

	LNS	FSIQ-2	BAPM A total	BAPM B total	PM	RM	LDT	Event-based PM	Time-based PM
LNS	-	0.26	0.10	−0.03	−0.33	−0.18	0.15	0.09	0.04
FSIQ-2	0.44^*^	-	−0.42^*^	−0.05	−0.24	−0.15	0.28	−0.13	0.48^*^
BAPM A total	0.03	0.25	-	−0.17	0.49^*^	0.19	0.11	0.16	0.16
BAPM B total	−0.04	0.40^*^	0.42^*^	-	−0.12	−0.31	−0.20	−0.27	−0.42^*^
PM	0.21	0.13	0.51^*^	0.19	-	0.59^**^	0.06	0.08	0.13
RM	0.08	0.11	0.46^*^	0.19	0.78^**^	-	0.23	−0.07	0.06
LDT	0.27	0.24	−0.09	0.02	0.05	0.10	-	0.05	0.03
Event-based PM	−0.03	−0.08	0.12	−0.07	0.14	−0.17	0.24	-	0.01
Time-based PM	−0.11	0.07	−0.47*	−0.18	−0.25	−0.13	0.16	−0.13	-

Older adult group results above central line, Young adults below. BAPM, brief assessment of prospective memory; PRMQ, prospective and retrospective memory questionnaire; RM, retrospective memory; and PM, prospective memory.

* *p* < 0.05;

** *p* < 0.01.

### Age-PM Paradox

#### LDT PM Performance

To evaluate LDT PM performance, the dependent variable was LDT PM accuracy as per other similar studies (e.g., Niedzwienska and Barzykowski, 2012). A one-way ANOVA was conducted to examine the effects of age group on LDT PM accuracy. There was significant main effect of age group, *F*(1,48) = 28.32, *p* < 0.001, $\eta_{p}{}^{2}$ = 0.37, with older adults performing worse (*M* = 0.46, *SD* = 0.27) than young adults (*M* = 0.78, *SD* = 0.15). This was a large effect size.

To evaluate LDT OT performance, the dependent variable was LDT OT accuracy. For OT accuracy, there was a significant main effect of age group, *F*(1,49) = 25.52, *p* < 0.001, $\eta_{p}{}^{2}$ = 0.32, with older adults performing worse (*M* = 0.77, *SD* = 0.10) than young adults (*M* = 0.88, *SD* = 0.05). This was a large effect size.

We also evaluated reaction time (RT) on the PM and OT trials. A one-way ANOVA was conducted to examine the effects of age group on PM RT. There was a significant main effect of age group, *F*(1,47) = 63.27, *p* < 0.001, $\eta_{p}{}^{2}$ = 0.57, whereby older adults (*M* = 776, *SD* = 74) responded significantly slower than younger adults (*M* = 625, *SD* = 59). This was a large effect size. For OT RT, there was also a significant main effect of age group, *F*(1,49) = 89.30, *p* < 0.001, $\eta_{p}{}^{2}$ = 0.65, whereby older adults (*M* = 752, *SD* = 51) responded significantly slower than younger adults (*M* = 630, *SD* = 41). This was a large effect size.

#### Ecological PM Performance

To examine ecological PM performance, the dependent variable was time- and event-based PM accuracy as per other similar studies (e.g., Shum et al., 2013). A 2 × 2 mixed ANOVA was conducted to investigate the main and interactive effects of ecological PM task type (time- vs. event-based) and age group on PM task accuracy. A significant main effect of age group was found, *F*(1, 45) = 18.07, *p* < 0.001, $\eta_{p}{}^{2}$ = 0.29, with young adults scoring higher than older adults. This was a large effect size. There was no significant main effect of PM task type, *F*(1, 45) = 0.09, *p* = 0.770, $\eta_{p}{}^{2}$ = 0.00, or interaction between PM task type and age group, *F*(1, 45) = 0.00, *p* = 0.965, $\eta_{p}{}^{2}$ = 0.00.

**Figure 1:**
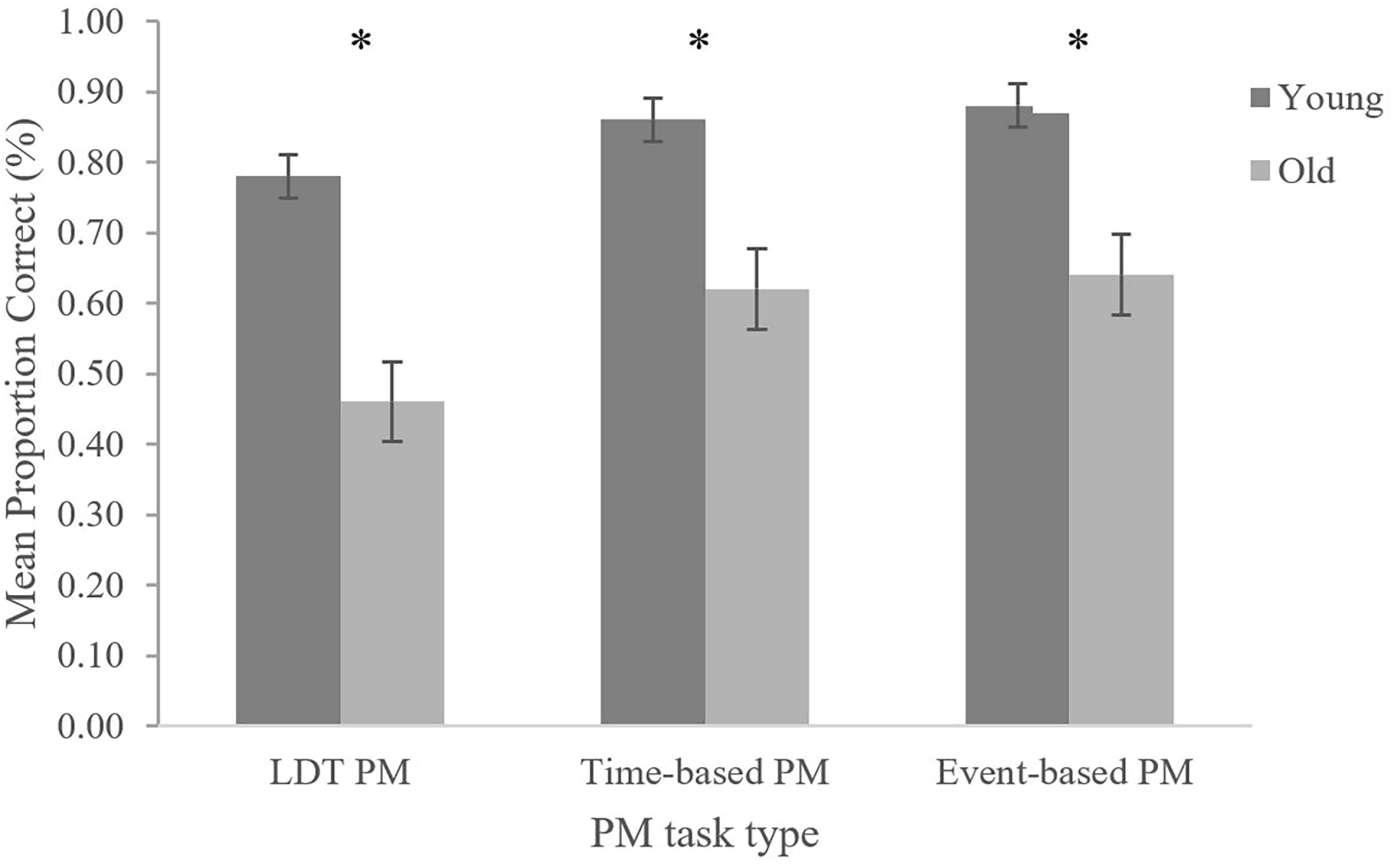
Correct PM performance in all PM tasks for both age groups. Error bars represent the SE. *statistically significant, p < 0.05.

Planned comparison revealed that older adults performed significantly worse on both time-based, *t*(34.67) = 2.50, *p* = 0.020, *d* = 0.72, and event-based PM, *t*(30.39) = 2.87, *p* = 0.007, *d* = 0.86, than young adults. These effect sizes were large. Moreover, planned comparisons revealed that both young and older adults did not perform significantly different on time-based compared to event-based PM (see Table 4). Lastly, a one-way ANOVA was conducted to examine the effects of age group on recipe OT accuracy. Results revealed that there were no significant differences between the age groups, *F*(1, 49) = 0.50, *p* = 0.484, $\eta_{p}{}^{2}$ = 0.01. See Table 5 for OT accuracies for all PM tasks (Figure 1).

**Table 4:** Planned comparisons on the PM Task accuracy.

	*t*	*df*	*p*	*d*
**Young vs. Old**				
Ecological time-based	2.50	34.67	0.020^*^	0.72
Ecological event-based	2.87	30.39	0.007^**^	0.86
LDT	5.09	32.87	0.000^***^	1.51
**Time- vs. event-based PM**				
Young	−0.28	23	0.78	−0.06
Old	−0.19	22	0.86	−0.04

PM, prospective memory; LDT, lexical decision task.

* *p* < 0.05;

** *p* < 0.01;

*** *p* < 0.001.

**Table 5:** Ongoing task accuracy and completion time on PM Tasks.

	Young	Old
	(*n* = 28)	(*n* = 23)
	*M (SD)*	*M (SD)*
**LDT**		
OT acc	0.87 (0.05)	0.77 (0.10)
**Recipe task**		
OT acc	0.73 (0.15)	0.70 (0.13)
Completion time	32:37 (5:02)	42:47 (10:54)

LDT, lexical decision task; OT acc, ongoing accuracy.

### Associations Between PM Measures

Correlational analyses were conducted between all PM tasks and with self-report PM measures. As shown in Table 3 there were no significant relationships between scores on the LDT PM and event-based and time-based PM tasks for young adults. For young adults, there was a significant medium negative correlation between BAPM A Total and time-based PM (*r* = −0.47). That is, for young adults, higher frequency of PM lapses was associated to lower time-based PM accuracy. Similarly, for older adults, there were no significant correlations between LDT PM task, event-based and time-based PM tasks. However, there was a significant medium negative relationship between BAPM B total and time-based PM (*r* = −0.42). That is, higher PM importance ratings were associated to lower scores on time-based PM accuracy.

## Discussion

The purpose of this study was to investigate the age-PM paradox by addressing several key methodological issues evident in previous research. Overall, strong age effects were found across all tasks, whereby older adults performed worse than young adults. Regarding correlational findings, less frequent self-report PM failures were associated with better time-based PM performance only for young adults. Meanwhile, for older adults, higher perceived importance of PM failures was associated with worse time-based PM performance. Nevertheless, due to the high functioning older adult sample obtained, it was expected that they would report less PM failures. For both age groups, there were no significant relationships between the LDT PM task, event-based and time-based PM tasks.

### Age-PM Paradox

Our first hypothesis was only partially supported. In line with previous research, compared to young adults, older adults performed significantly worse on the laboratory PM task, (Bisiacchi et al., 2008; Altgassen et al., 2010). However, older adults also underperformed in comparison to young adults on the ecological PM tasks (time-based and event-based PM). The finding that older adults did not perform better than young adults on the ecological tasks is not consistent with the age-PM paradox. These findings may suggest that the age-PM paradox does not exist. That is, regardless of the nature of the PM tasks, older adults still perform worse than younger adults.

Cognitive processes such as learning, memory, and executive functions that rely on the prefrontal and medial temporal cortex functions show considerable decline with age (Fisk and Sharp, 2004; Burke and Barnes, 2006; Luo and Craik, 2008; Cona et al., 2012). Similarly, at the neuro-anatomical level, there is evidence that PM is heavily reliant on both prefrontal systems (Brodmann’s Area 10; Burgess et al., 2001) and the medial temporal lobe (Gordon et al., 2011). Consequently, robust age effects should be expected when comparing PM performance on tasks that involve these kinds of functions between younger and older age groups.

Although a popular explanation for the age–PM paradox is that older adults are efficient with external reminders in everyday life (Phillips et al., 2008; Ihle et al., 2012), several studies have found that reminder use does not account for age effects in event and time-based naturalistic tasks (Niedzwienska and Barzykowski, 2012). The results of this study are important as it addressed some major limitations within the existing literature. For example, administering both laboratory and ecological PM tasks for both older and younger individuals in the same study, and using an ecological PM task that has more than one or two PM cues, and without external reminders. Hence, while the age-PM paradox is an interesting phenomenon; our findings suggest that it may not occur if laboratory and ecological tasks are designed to be more comparable. That is, the ecological nature of a task by itself may not guarantee or is not enough to reveal an age-PM paradox.

The second hypothesis was not supported in that we did not find a task type main effect for both young and older adults, that is, performance did not significantly differ between the time-based and event-based PM ecological tasks. Event-based PM tasks typically uses less cognitive resources for completion than time-based PM tasks because the latter lack external cues to prompt the intended action and thus require more self-initiated processing such as time monitoring (Einstein et al., 1995; Kvavilashvili and Fisher, 2007).

The added complexity in our ecological task in combination with a relatively high functioning older adults’ sample, might have masked the traditional time- vs. event-based task differences. Others have also postulated that the distinction into time- and event-based PM tasks in everyday life may not be as apparent as once imagined (cf. Schnitzspahn et al., 2018). In fact, some studies have found that older adults performed better on time-based tasks or did not show greater age-related declines on time-based PM tasks (d’Ydewalle et al., 1999; Rendell and Craik, 2000; Haines et al., 2020). Thus, the lack of significant differences between the two task type results may be due to similarly high cognitive demands of the two PM tasks.

### Self-Reported PM

The third hypothesis that there would be significant associations between the self-reported PM measures (BAPM A and PRMQ PM subscale) was supported. We found significant positive correlations between the BAPM A and PMRQ PM, with the young adults yielding a slightly larger correlation than the older adults. No significant relationships, nevertheless, were found between BAPM B and the PMRQ subscales. There were also significant positive correlations between BAPM A and PMRQ RM subscale, and BAPM A and BAPM B only for young adults. These results support the concurrent validity of the BAPM A and PMRQ PM subscale in measuring self-reported PM failure frequency in both age groups. In addition, measuring frequency of failure (BAPM A and PMRQ PM) may be conceptually different from perceived importance (BAPM B), thus, the absence of relationships. Given this finding, measuring both aspects of self-reported PM is important because it can provide additional information about PM functioning in individuals.

The fourth hypothesis that the self-reported PM measures should reflect the age-PM-paradox was partially supported. We found that on the self-reported PM measures, older adults reported less frequent PM failures (Part A) but rated PM failures as more problematic (Part B), meanwhile the reverse was true for young adults. That is, they reported more frequent PM failures, but failures were rated as less problematic. On the PRMQ, young adults reported significantly more PM failures compared to older adults, with no significant differences on RM errors. This is contrary with previous research showing no difference in the self-reported memory errors between the young and older adults on the PRMQ (Smith et al., 2000; Crawford et al., 2003). However, the different patterns of correlations for these self-reported PM measures may indicate that severity of potential PM failures does not necessarily relate to frequency of failures, especially for highly functioning individuals. For example, when perceived importance is high, older adults may make extra effort using external reminders to ensure the completion of those tasks. Moreover, the questionnaires mostly refer to naturalistic PM tasks. Thus, it can also be interpreted that the less reported PM failures can also provide some support for the age-PM paradox (i.e., less PM failures in naturalistic settings).

Previous studies have found that busy people experience more PM failures and subsequently rate their PM as poor (Uttl and Kibreab, 2011). For young adults, more frequent forgetting could result from the lower perceived importance of such tasks due to busier lives. While for older adults, PM failures are more salient and have more severe consequences than other memory problems such as semantic memory failure (Henry et al., 2004). Therefore they would show better insight in PM failures in daily life and rated them as more important (Ossher et al., 2013). For example, medication mismanagement due to PM failure may result in significant health consequences for older adults. Our findings on the BAPM B were consistent with this notion, as older adults rated PM failures as having more significant consequences, while the reverse was true for young adults. It is worthy to note that our older adult group is a high functioning group, with more years of education (albeit not statistically significant) and higher IQ than the young adult group. Thus, they may be very active in their retirement and value cognitive stimulation.

#### Self-Reported PM and Behavioral PM Performance

We were also interested in examining whether there were significant associations between self-reported PM and PM performance. Although no significant correlations emerged between the tasks, younger adults still outperformed older adults on all three PM tasks. Thus, these tasks appear to be sensitive enough to detect aging effects since no floor or ceiling effects were present. The absence of significant relationship between the laboratory-based PM tasks and ecological PM tasks suggest that they may be tapping into different aspects of PM. For example, Schnitzspahn et al. (2018) examined the age-PM paradox, while distinguishing between experimenter-assigned and self-assigned PM tasks. They found that age benefits were only observed for naturalistic time-based tasks, but not for participants’ own self-assigned time-based task. The authors concluded that these age benefits for naturalistic PM tasks may have been in part related to the dominant use of experimenter-generated naturalistic time-based PM tasks in previous studies.

The current findings corroborates with another study using a simpler measure of naturalistic PM (requesting an envelope from the examiner at the end of experiment) and a computerized shopping task also found a lack of relationship between their tasks in their healthy older adults’ group (Lee et al., 2018). They found medium sized relationships between their PM tasks, only among their older adults who reported subjective memory decline, but not in their healthy controls. Moreover, the association between performance on their shopping task and cognitive tasks were all small in effect size for the healthy controls. Thus, our findings are not surprising, since stronger relationships between cognitive variables and PM performance are more common among clinical populations (Thompson et al., 2010).

We only found a significant negative correlation between the BAPM B and time-based PM performance for older adults – the more important older adults rated PM failures, the worse they performed on time-based PM task. These results are counterintuitive, but their perceived importance of PM tasks and associated anxiety may have hindered their ability to perform. There were no other significant associations between behavioral and self-reported PM. We expected poor associations between self-reported PM and laboratory-based task as they typically do not reflect activities in everyday life. Some studies show that time-based PM is more strongly associated with instrumental activities in everyday functioning (Tierney et al., 2016), while other studies suggest that event-based PM shows stronger associations with medication management (Woods et al., 2014). Thus, seeing that our sample of older adults were highly functioning, it makes sense that we only found associations on time-based PM.

The current findings are consistent with evidence from several previous studies showing that the associations between objective memory performance and self-reported memory assessments among older adults are modest at best (Zeintl et al., 2006; Uttl and Kibreab, 2011; Crumley et al., 2014). Although there are limitations with self-reported measures, they shed light on an individual’s own perspective and awareness of how frequently PM lapses occur as well as the significance of these lapses. Particularly for older adults, this would be useful to help with strategies to preserve functioning. Together, these findings caution against relying on self-reported measures as a proxy for objective assessments of PM.

Overall, the findings of this study provide evidence that age-related PM declines are strong across ecological and laboratory PM tasks. Interestingly despite the robust age effects that are present, the absence of associations between our ecological and laboratory PM tasks suggests that both task types may be capturing different aspects of PM. Importantly, the present study adds to the body of evidence regarding the discrepancies between objective PM performance and subjective PM.

### Limitations and Future Research

The generalizability of these findings are limited as the sample consisted of highly educated and high functioning individuals. Thus, future studies should consider collecting data on a more diverse group of older and younger adults so that stronger conclusions can be drawn about the effect of aging across laboratory and ecological PM tasks. It could be argued that the relatively higher complexity of our ecological PM task did not accurately reflect PM requirements in daily life and therefore hindered the age-related advantage. For example, in everyday life constant monitoring in a confined timespan with multiple tasks may be unrealistic during daily life in retirement. This is more likely to occur for younger adults who lead busier lives. However, this explanation is also unlikely due to the older adults group having more years of education and higher IQ.

We also did not include a time-based task for our laboratory PM task. This did not enable us to directly compare the time-based tasks between laboratory and ecological tasks. Although the complex nature of PM in everyday life would make embedding a time-based task in a simple laboratory task quite difficult. Alternatively, the age-PM paradox may not have emerged because the laboratory PM task and the ecological PM were not equal in difficulty. That is, while the ecological PM task, we used in our study required participants to store the event-based PM cue in memory as well as simultaneously monitor the time-based cue, participants only needed to handle the event-based PM cue in the laboratory task. Consequently, older adults were also found to be impaired on the ecological PM task. Future studies would benefit from employing an equal time-based task in their laboratory PM and including time-monitoring measures. It would also be helpful to include other EF measures such as inhibitory control and task switching to further clarify the relationship between the PM processes in our ecological task.

## Conclusion

In summary, the current findings support the view that PM declines with age and decrements in performance can be assessed using behavioral PM tasks. The lack of age-related differences between the ecological event and time-based PM task may suggest that the distinction between ecological time- and event-based PM tasks may not be practically meaningful in everyday life. However, despite this, evidence of age-related decline was still robust across all the PM tasks. These findings provide theoretical advance in explicitly considering the differing cognitive demands and age-effects, particularly time-based PM in ecological settings. It also makes a novel contribution and adds important evidence about some of the neglected mechanisms (task complexities and type) contributing to the long-standing age-PM paradox.

## Data Availability Statement

The raw data supporting the conclusions of this article will be made available by the authors, without undue reservation.

## Ethics Statement

The studies involving human participants were reviewed and approved by Griffith University Ethics Committee. The patients/participants provided their written informed consent to participate in this study.

## Author Contributions

YK and DS designed the study and analyzed data. YK wrote the manuscript. All authors contributed to the article and approved the submitted version.

### Conflict of Interest

The authors declare that the research was conducted in the absence of any commercial or financial relationships that could be construed as a potential conflict of interest.
